# Surgical management of a massive congenital hemangioma of the tongue in an infant: A rare case report

**DOI:** 10.1002/ccr3.4909

**Published:** 2021-10-06

**Authors:** Krishan Sarna, Martin Kamau, Symon Guthua

**Affiliations:** ^1^ Department of Human Anatomy University of Nairobi Nairobi Kenya; ^2^ Division of Oral and Maxillofacial Surgery Department of Oral and Maxillofacial Surgery, Oral Pathology and Oral Medicine University of Nairobi Nairobi Kenya

**Keywords:** congenital hemangioma, hemangioma, massive hemangioma, super‐selective embolization, surgical management, tongue hemangioma

## Abstract

Hemangiomas of the tongue may reach a massive size resulting in functional compromise. Surgical resection, despite the risk of hemorrhage, may be the only option if conservative measures such embolization are futile in reducing its size.

## Graphical abstract

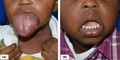

## 1 INTRODUCTION

Lesions involving the tongue may present with life threatening complications such as airway compromise and risk of severe hemorrhage. This paper reports the surgical management of a non‐involuting massive congenital hemangioma of the tongue in an infant presenting with difficulty breathing, feeding and inability to close the mouth.

Hemangiomas are common congenital head and neck anomalies characterized by rapidly proliferating endothelial cells that line blood‐filled spaces, having an incidence of 3%–10% at 1 year of age but disappear completely by 5 years of age in about 50%–80% of cases.^1^, ^2^ While 60%–70% of hemangiomas are found within the head and neck region, oral hemangiomas are rare, frequently involving the tongue, lips, buccal mucosa, palatal mucosa, and jaw bones. They appear as a stain or nodule that is either smooth, lobulated, sessile, or pedunculated that varies from deep red to a purple color depending on its size and depth in tissue.^3^, ^4^, ^5^ The condition is more frequent among premature low‐birth–weight infants, gestational hypertension, twins, and affects females three times more than males. The International Society for the Study of Vascular Anomalies (ISSVA) classifies these as benign vascular tumors either of the infantile or congenital variety based on time of presentation and behavior of the lesion.^6^

Hemangiomas of the tongue usually present as small lesions less than 3–5 cm in size. However, giant lingual hemangiomas involving up to a half or two‐thirds of the tongue have been reported previously, posing a significant risk to patient which may considerably deteriorate their quality of life.^7^, ^8^ It is crucial to note that these reports involved management of hemangiomas in older patients and to the best of our knowledge this is the first report that addresses the surgical management of such a complex case in an infant. Early diagnosis and treatment of such lesions is vital in preventing catastrophic complications such as traumatic hemorrhage, difficulty in breathing, feeding, speech, delay in linguistic development and mandibular dysmorphogenesis.^9^

Hemangiomas usually do not require any treatment due to their benign nature and high rate of complete involution over time. However, in cases where the lesion fails to involute despite conservative measures or is extremely large and poses an imminent threat to life, surgical intervention may be the only treatment of choice despite the inherent risk of potentially fatal intraoperative hemorrhage.^10^, ^11^, ^12^, ^13^ We therefore aim to describe the successful management of an infant presenting with a massive hemangioma involving two‐thirds of the tongue with a potential for fatal complications had surgical intervention not been carried out.

## 2 CASE PRESENTATION

**FIGURE 1:**
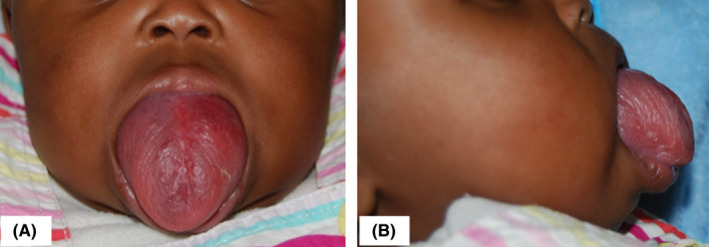
Anterior view (A) and a lateral view (B) of the lesion involving the tongue on first presentation prior to treatment

We report a case of a 6‐month old girl referred to the Nairobi hospital with a massive, progressively growing congenital mass in the oral cavity. A thorough medical history revealed no co‐morbidities with all vital signs within normal limits. Upon physical examination, the patient was found to have difficulty in breathing, difficulty feeding and inability to close the mouth. Intra oral examination revealed a large swelling involving more than two‐thirds of the tongue, most of which lay outside the oral cavity (Figure 1). The lesion was a deep red‐purple color, soft on palpation, non‐tender and normal in temperature with no appreciable thrills but blanched on application of pressure. Examination of the surrounding structures was unremarkable.

**FIGURE 2:**
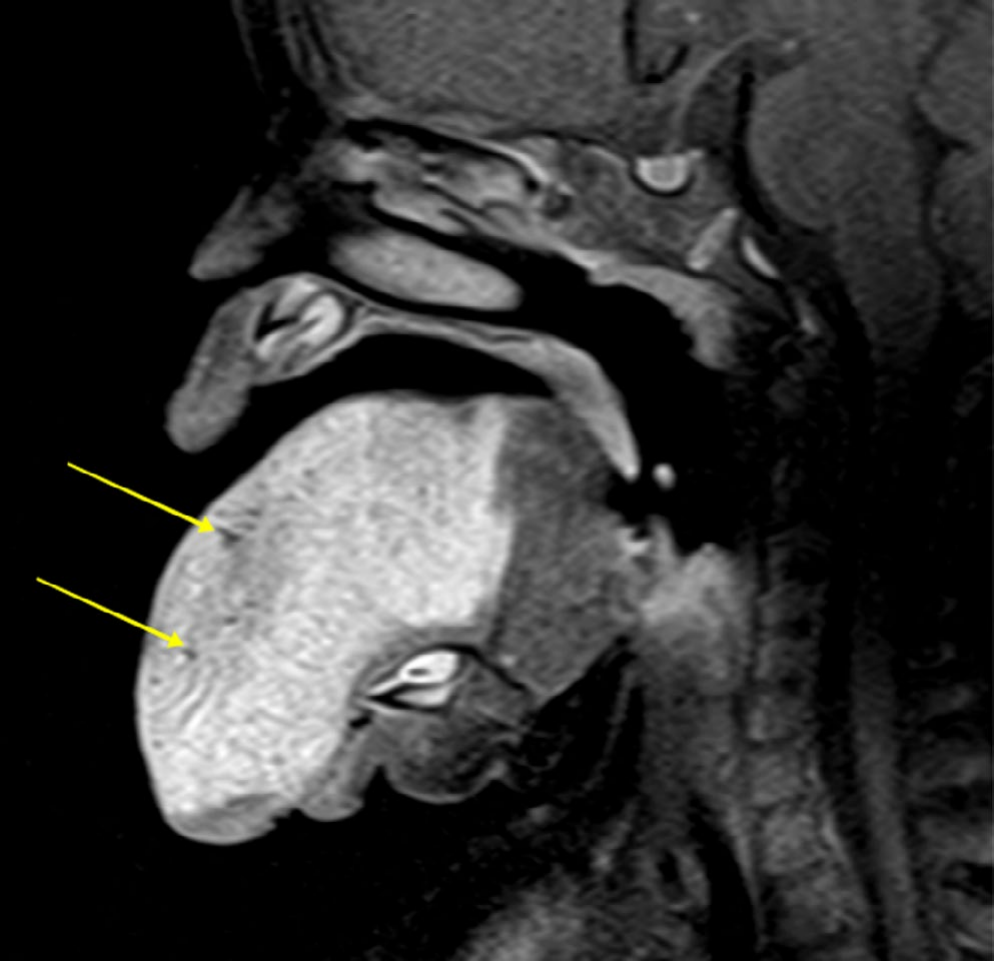
T2‐weighted contrast enhanced MRI sagittal section showing a hyperintense mass on the tongue. Small foci (yellow arrows) can be seen within the lesion corresponding to flow voids

Magnetic Resonance Imaging (MRI) with gadolinium revealed a well‐circumscribed soft tissue mass measuring 3.9 cm × 4.1 cm × 5.8 cm. It involved most of the tongue except for the base and extended to both lateral borders, while inferiorly, involved the entire thickness of the tongue to its ventral surface. The mass was hypointense on T1‐weighting and was found to be hyperintense on T2‐ weighting. Multiple low signal foci were noted which were compatible with flow voids (Figure 2). Digital subtraction angiography revealed the feeder vessels to be anomalous branches of the lingual arteries. These findings, coupled with blood collection in the interior of the lesion, informed the diagnostic hypothesis of a congenital lingual hemangioma.

**FIGURE 3:**
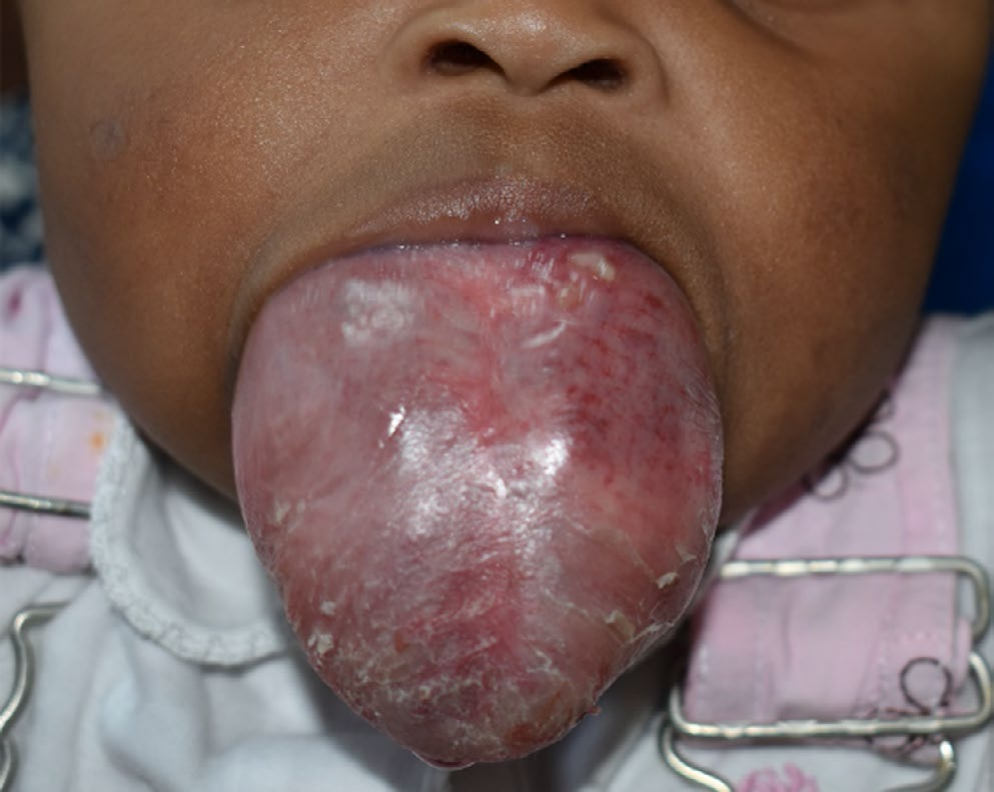
Appearance of the lesion after 1 year of observation. The lesion has increased in all dimensions and now occupies majority of the oral cavity. The surface appears dry and flaking of the superficial layers of mucosa can be seen

**FIGURE 4:**
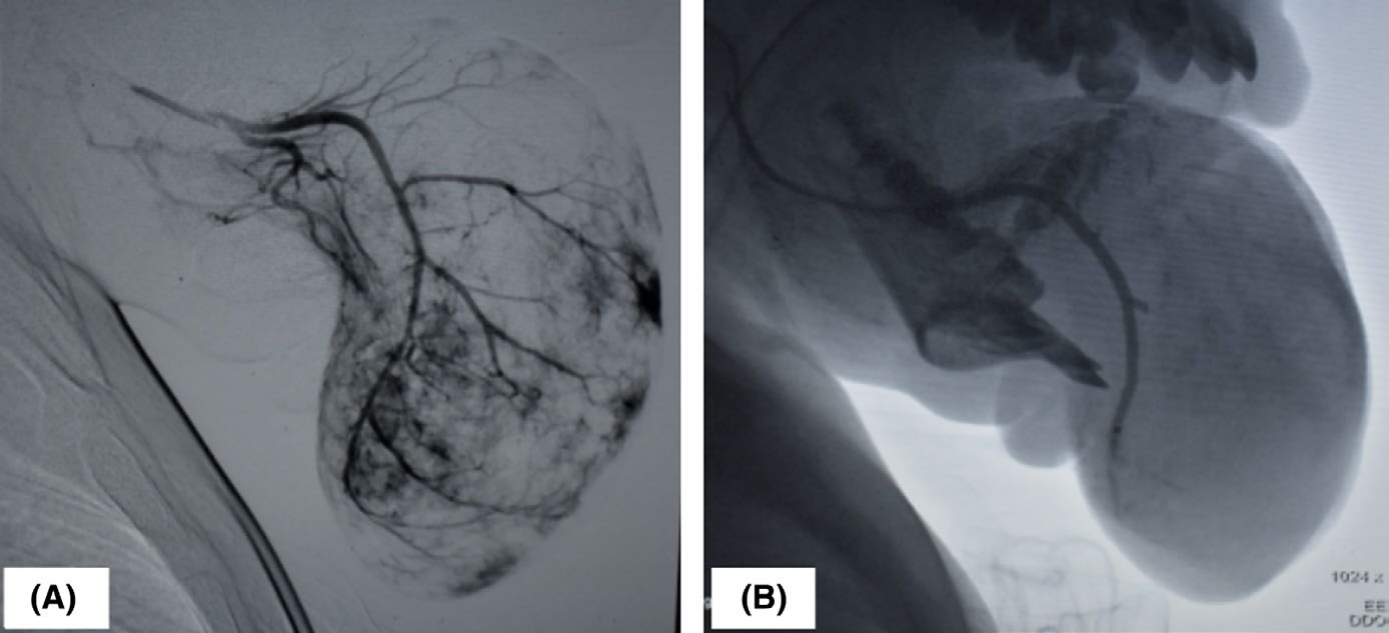
Digital subtraction angiogram of the left lingual artery prior to embolization (A) and immediately after embolization (B). Majority of the feeder vessels have been successfully occluded and blood supply to the lesion appears to be reduced

The lesion was monitored closely over a period of 1 year with the expectation of improvement by involution, however, the lesion showed no signs of reducing in size and in fact enlarged over this duration (Figure 3). After a multidisciplinary discussion, immediate embolization of the feeder vessels and subsequent surgical resection of the lesion was proposed in order to improve the quality of life for the patient and prevent fatal airway obstruction. Embolization of the lingual arteries (LA’s) was performed through a transfemoral approach. Using the bilateral lingual arteriograms, the feeder vessels from the right and left LA’s were selectively embolized as close to the lesion as possible using absorbable gelatin sponge particles (Gelfoam, 100–200 μm) under fluoroscopic guidance. Post‐embolization angiograms of the LA’s were performed to confirm if all feeder vessels had been occluded (Figure 4).

**FIGURE 5:**
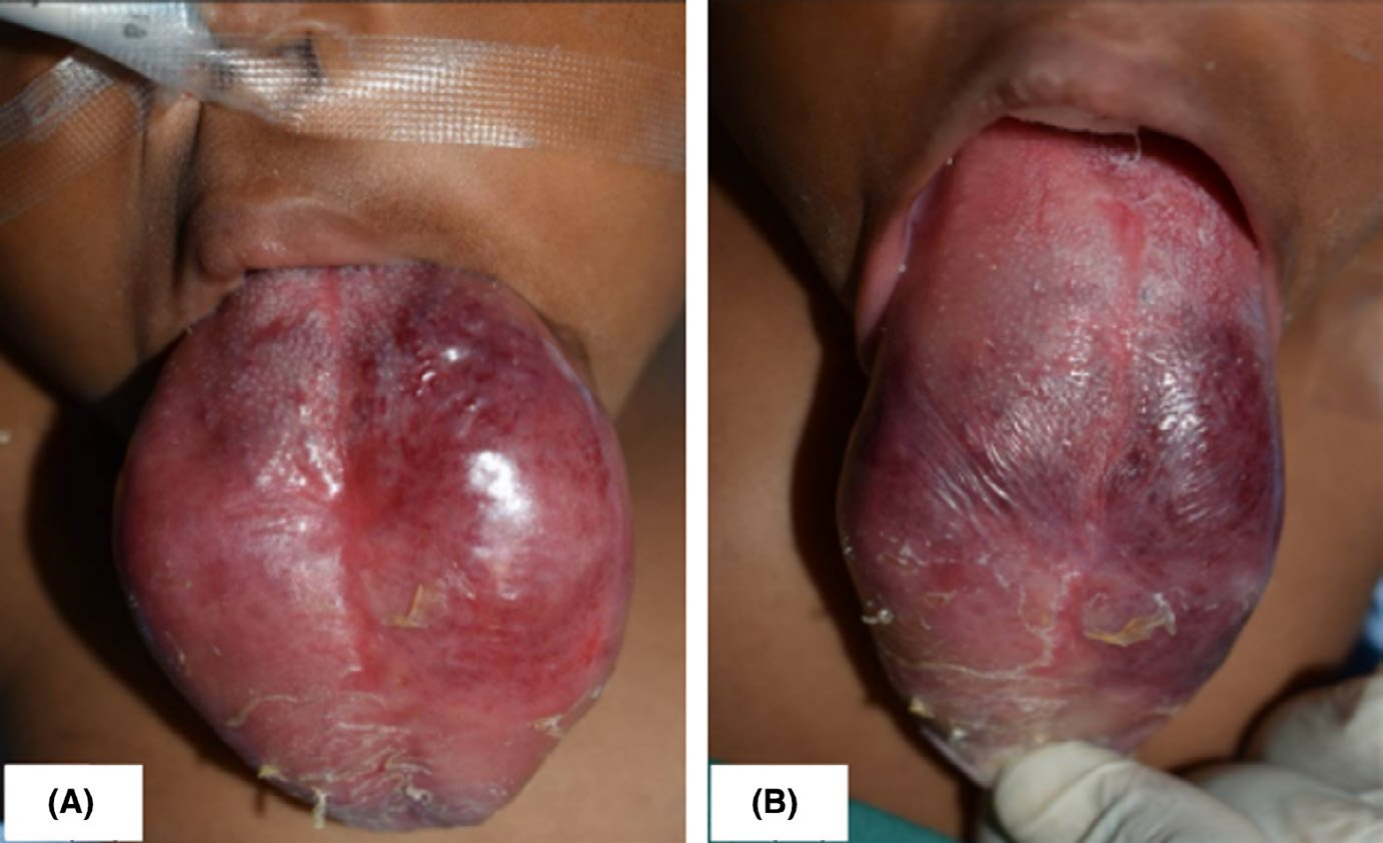
Clinical appearance of the lesion prior to embolization (A) compared to its appearance immediately after embolization (B). Even though the lesion appears to be reduced in size, feeding was still a major challenge

**FIGURE 6:**
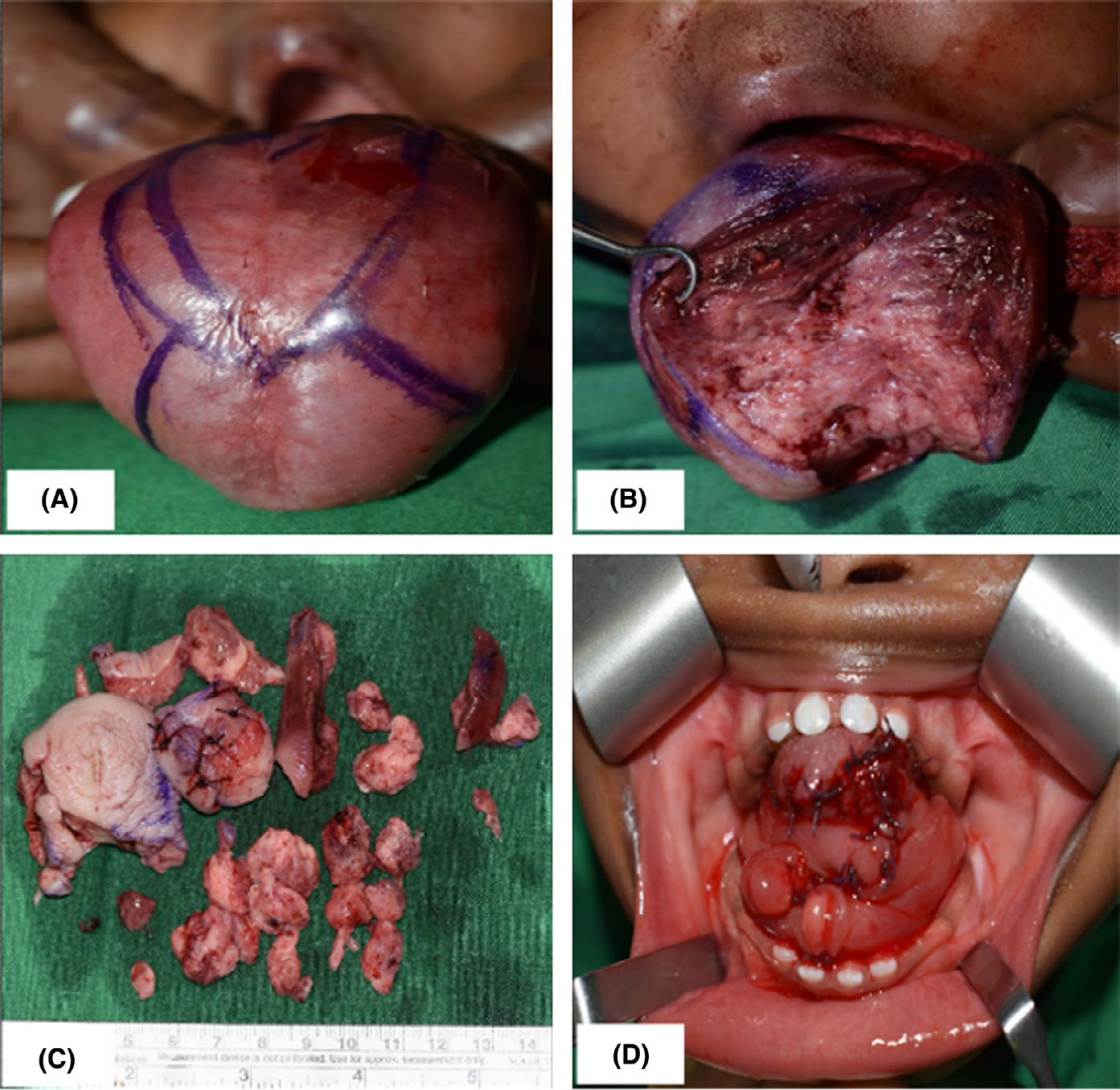
Surface markings on the tongue using indelible ink prior to dissection (A). Excision of the transverse and vertical lesion and reduction of the anterior two‐thirds of the tongue (B). Excised specimen from the tongue (C). Appearance of tongue immediately after suturing (D)

One month post embolization, a color change from deep red/purple to a lighter shade was noted and the lesion seemed to have reduced slightly in size as shown by its surface having mucosal folds as opposed to appearing stretched and shiny prior to embolization (Figure 5). Despite this, feeding and airway management were still a major problem for the patient. A month later, naso‐tracheal intubation under endoscopic guidance was performed and a modified key hole surgical technique was used to reduce the bulk of the lesion (Figure 6). A full thickness elliptical wedge incision was made on the dorsum of the tongue using electrocautery, accompanied by two diverging incisions on the anterior portion of the tongue. Hemorrhage was controlled by compression of the posterior aspect of the tongue using moist gauze after which excess tissue from the central and anterior portion of the tongue was excised. Suturing was done in various planes using vicryl 3.0 after attaining the best fitting of the tongue into the oral cavity. In the immediate post‐operative period, there was slight swelling of the tongue which resolved in a few days.

**FIGURE 7:**
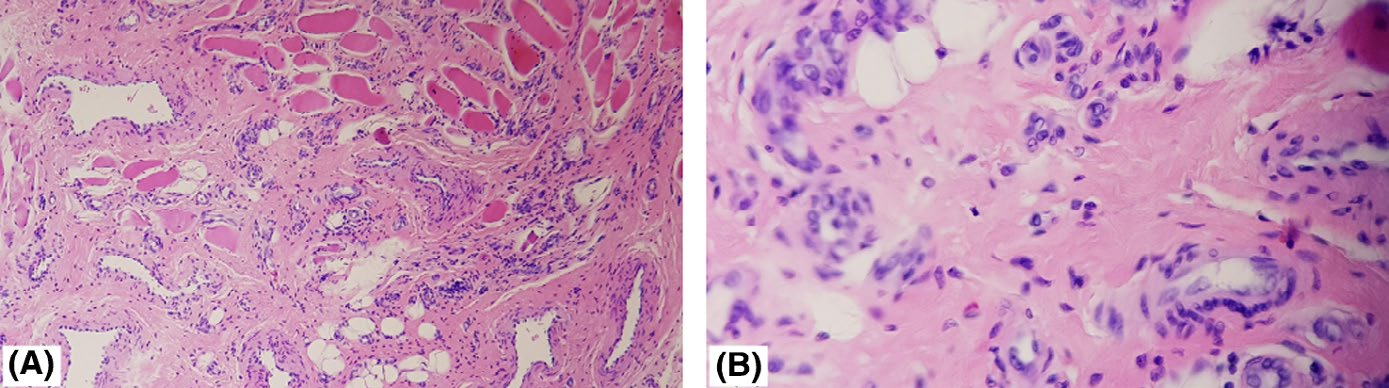
Capillary hemangioma of the tongue: Presence of both thick and thin walled vascular channels lined by endothelial cells without anaplastic features (hematoxylin and eosin, magnification ×10 (A) and ×40 (B))

**FIGURE 8:**
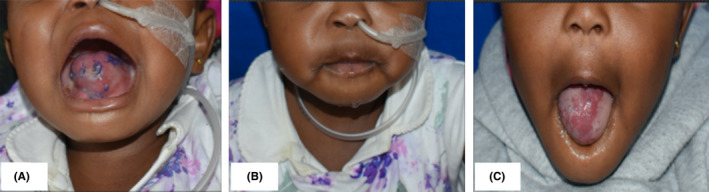
Appearance of the tongue 1 week after surgery (A) with the tongue satisfactorily contained in the mouth, note the nasogastric tube (B). Appearance 8 months after surgery (C) with restored tongue form and function (protrusion)

**FIGURE 9:**
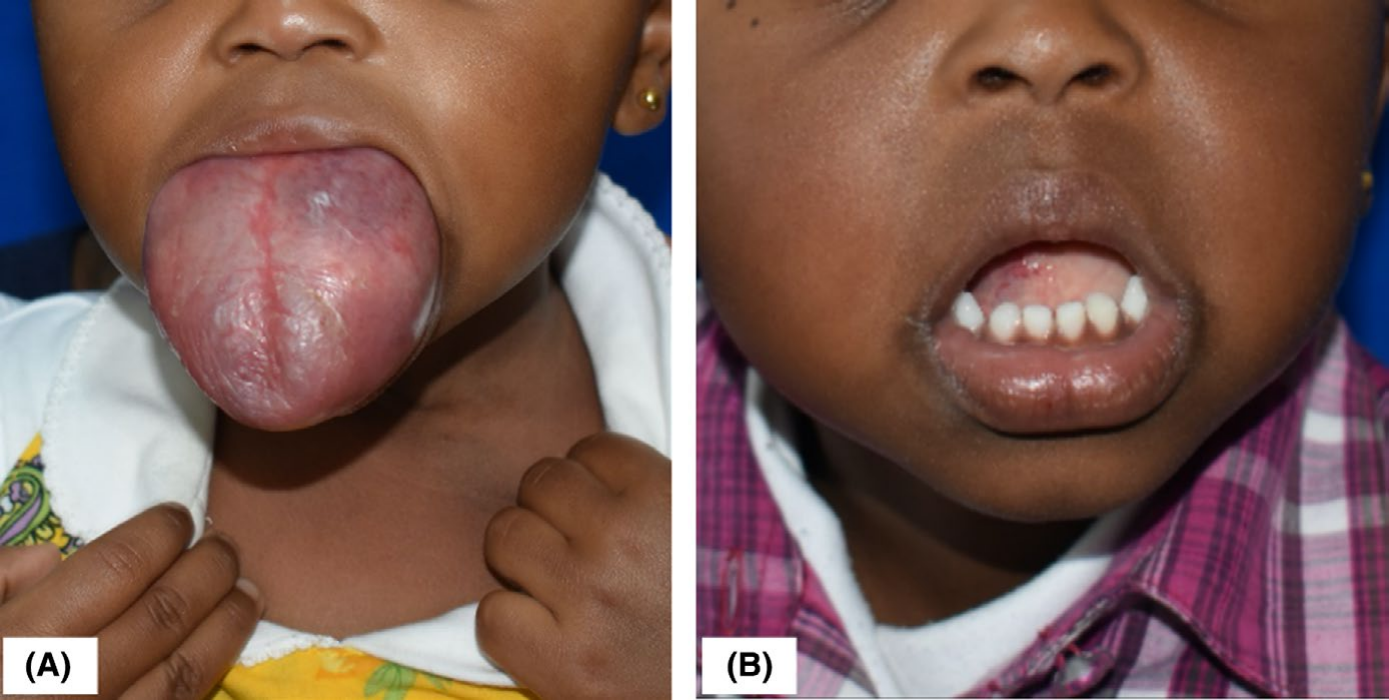
A comparison of the tongue before surgery (A) and at medium term of 1 year and 9 months after surgery (B)

Postoperative hospital stay was uneventful with no medical or surgical complications. The patient was fed via a nasogastric tube for a period of 5 days after which it was removed and oral feeding begun. The patient was discharged on the 8^th^ day after surgery. Histology of the tissue specimen revealed the diagnosis to be a capillary hemangioma (Figure 7). At the final follow‐up visit, the tongue had healed entirely and could fit within the mouth comfortably. The patient showed marked improvement in speech and swallowing functionality with no other complaints (Figures 8 and 9).

## 3 DISCUSSION

Hemangiomas deserve special attention and consideration, not because of their rarity of occurrence but due to the potential of life‐threating complications such as airway obstruction and hemorrhage. Similar to hemangiomas in other anatomical sites, hemangiomas of the tongue occur at birth or shortly after and grow rapidly up to 6 months of age after which they spontaneously involute.^14^ The present lesion demonstrated deviation from expected course and instead continued to increase significantly in size with no signs of involution.

The etiopathogenesis of congenital hemangiomas remains unknown. However, theories such as the placenta theory, metastatic theory, progenitor cell theory and the extrinsic factor theory attempt to describe the development of infantile hemangiomas.^15^ In rare cases, hemangiomas may be found in patients with Sturge‐Weber syndrome, Rendu‐Osler Weber syndrome and Blue rubber bleb syndrome.^16^ In majority of cases a diagnosis of a hemangioma is readily arrived at using a characteristic history of the lesion and local examination.^17^ Radiographic imaging techniques such as Computed Tomography (CT) or MRI are crucial in volumetric analysis of the lesion and assessment of surrounding structures especially when the lesion is large enough to cause airway compromise or displacement of vital structures.^12^ Digital subtraction angiography provides an additional host of information regarding the nature of involved vasculature, intralesional flow rate and is a critical aid during selective embolization.

Management of hemangiomas depend upon factors such as age, size, location, involved vasculature and response to any previous treatment strategies. Numerous conservative approaches may be implemented prior to opting for surgical intervention including corticosteroid therapy, beta blocker therapy, sclerotherapy, cryotherapy and laser therapy.^18^, ^19^ In the present case the techniques described above could not be used owing to the fact that the lesion was enlarging at a rapid pace and may have compromised the patient's airway before these conservative measures could produce a substantial reduction in the size of the lesion. Therefore, the decision to embolize the feeder vessels and surgically resect the lesion was made.

Large vascular malformations usually respond well to selective embolization, reducing the lesion's size significantly to avert life‐threatening complications.^20^ This may even reduce the size of the lesion to a point where the need for a surgical procedure is unnecessary.^21^ However, also embolization in this case did not have the expected results, hence necessitating surgical intervention. Surgical resection of vascular lesions involving the tongue is a dangerous and precarious procedure that may lead to substantial intraoperative hemorrhage and possibly fatality. The tongue is perfused bilaterally by the LA’s which are large caliber branches originating from the external carotid artery and therefore hemorrhage from a vascular lesion involving this region can quickly prove to be lethal.^22^ In this case, the tender age in association with the size and location of the lesion heightened the risk significantly. However, the danger of airway compromise significantly outweighed the risk of hemorrhage during surgery which determined the decision to continue with the treatment plan.

A modified key hole resection technique was specifically devised to achieve adequate reduction in both transverse and antero‐posterior dimensions of the tongue that would allow it to fit in the oral cavity but yet ensure that functionality remained intact.^23^ Intraoperative hemorrhage was encountered (200 cc) despite the pre‐operative embolization but was adequately controlled with bimanual compression of the posterior aspect of the tongue and local control with ligation of the offending vessels. Half a unit (250 cc) of packed red blood cells were transfused to compensate for the blood loss. The reason for the hemorrhage could have been attributed to incomplete embolization of all the feeder vessels or presence of a collateral blood supply. Noteworthy, the patient did not present with further complications. After 1 year of follow‐up, the patient has normal swallowing and respiratory function with better speech articulation.

## 4 CONCLUSION

Hemangiomas are a benign proliferation of endothelial cells that are common in the head and neck region. In the oral cavity, the lesion may involve the tongue and reach a size that can cause airway embarrassment and cause significant functional impairment. Surgical intervention, despite the inherent risk of fatal intraoperative hemorrhage, may be the only treatment of choice if other conservative measures do not produce the intended results or the rapid expansile nature of the lesion causes an imminent threat to life.

## CONFLICT OF INTEREST

No conflict of interest to declare.

## AUTHOR CONTRIBUTIONS

Krishan Sarna: Collected the data and wrote the findings of this manuscript. Dr. Martin Kamau: Provided supervision and guidance during preparation of the manuscript and approved the final version of the manuscript. Professor Symon Guthua: Performed the surgery in the case described, supervised the development of the manuscript and approved the final version.

## ETHICAL APPROVAL

The manuscript was prepared according to standard publication ethical guidelines.

## CONSENT

The authors confirm that a signed consent was obtained from the patient prior to publication.

## Data Availability

The data that support the findings of this study are available on request from the corresponding author. The data are not publicly available due to privacy or ethical restrictions.

## References

[1] Van Aalst JA, Bhuller A, Sadove AM. Peadiatric vascular lesions. J Craniofac Surg. 2003;14:566‐583. 12867875 10.1097/00001665-200307000-00032

[2] Shpitzer T, Noyek AM, Witterick I, et al. Noncutaneous cavernous of the head and neck hemangiomas. Am J Otolaryngol. 1997;18:367‐374. 9395011 10.1016/s0196-0709(97)90055-7

[3] Gombos F, Lanza A, Gombos F. A case of multiple oral vascular tumors: the diagnostic challenge on haemangioma still remain open. Judic Stud Inst J. 2008;2(1):67‐75.

[4] Dilsiz A, Aydin T, Gursan N. Capillary hemangioma as a rare benign tumor of the oral cavity: a case report. Cases Journal. 2009;9(2):8622. 10.4076/1757-1626-2-8622 PMC2827094 20181211

[5] Greene LA, Freedman PD, Friedman JM, Wolf M. Capillary hemangioma of the maxilla. A report of two cases in which angiography and embolization were used. Oral Surg Oral Med Oral Pathol. 1990;70(3):268‐273. 2216353 10.1016/0030-4220(90)90138-i

[6] Dasgupta R, Fishman SJ. ISSVA classification. Semin Pediatr Surg. 2014;23(4):158‐161. 25241091 10.1053/j.sempedsurg.2014.06.016

[7] Kamala K, Ashok L, Sujatha G. Cavernous hemangioma of the tongue: a rare case report. Contemp Clin Dent. 2014;5(1):95. 24808705 10.4103/0976-237X.128680 PMC4012128

[8] Agarwal M, Agarwal L, Mathur V. Hemangioma of tongue. AME Case Rep. 2018;2(1):11. 30264007 10.21037/acr.2018.03.04 PMC6155592

[9] Zheng JW, Zhou Q, Yang XJ, et al. Treatment guideline for hemangiomas and vascular malformations of the head and neck. Head Neck. 2010;32:1088‐1098. 19924783 10.1002/hed.21274

[10] Nguyen HP, Pickrell BB, Wright TS. Beta‐blockers as therapy for infantile hemangiomas. Semin Plast Surg. 2014;28(2):87‐90. 25045334 10.1055/s-0034-1376259 PMC4078206

[11] Zhang L, Zheng JW, Yuan WE. Treatment of alarming head and neck infantile hemangiomas with interferon‐α2a: a clinical study in eleven consecutive patients. Drug Des Devel Ther. 2015;9:723‐727. 10.2147/DDDT.S67682 PMC4324326 25678777

[12] Bonet‐Coloma C, Mínguez‐Martínez I, Palma‐Carrió C, Galán‐Gil S, Peñarrocha‐Diago M, Mínguez‐Sanz JM. Clinical characteristics, treatment and outcome of 28 oral haemangiomas in pediatric patients. Med Oral Patol Oral Cir Bucal. 2011;16(1):e19‐22. 20711165 10.4317/medoral.16.e19

[13] Stuepp RT, Scotti FM, Melo G, Munhoz EA, Modolo F. Effects of sclerosing agents on head and neck hemangiomas: a systematic review. J Clin Exp Dent. 2019;11(11):e1033‐e1044. 31700578 10.4317/jced.56143 PMC6825735

[14] V P, Puppala N, Deshmukh SN, B J, S A. Cavernous hemangioma of tongue: management of two cases. J Clin Diagn Res. Published Online 2014;8(10):ZD15‐ZD17. 10.7860/JCDR/2014/10216.5005 PMC4253281 25478463

[15] Lo K, Mihm M, Fay A. Current theories on the pathogenesis of infantile hemangioma. Semin Ophthalmol. 2009;24(3):172‐177. 19437354 10.1080/08820530902805438

[16] Khanduri S, Agrawal D, Varshney G, Singh N. Haemangioma of tongue: a rare case report. J Oral Maxillofac Radiol. 2015;3:25‐27.

[17] Shrestha AL, Paudel SB. Lingual cavernous hemangioma in a Nepalese boy—‘A Difficult Associate!!!’. J Surg Case Rep. 2018;2018(10):rjy283. 30370045 10.1093/jscr/rjy283 PMC6198281

[18] Satterfield KR, Chambers CB. Current treatment and management of infantile hemangiomas. Surv Ophthalmol. 2019;64(5):608‐618. 30772366 10.1016/j.survophthal.2019.02.005

[19] Guthua SW, Macigo FG, Wakoli KA. Combined modality approach in the management of circumoral haemangiomas with emphasis on use of Absolute Ethyl Alcohol. Afr J Oral Health Sci. 2011;6(1):5‐11.

[20] Kamata M, Aramaki‐Hattori N, Okabe K, et al. Embolization of congenital hemangioma with severe hemorrhage. J Pediatr Surg Case Rep. 2021;65:101772.

[21] Schwartz DN, Kellman RM, Cacayorin ED. Treatment of a lingual hemangioma by superselective embolization. Arch Otolaryngol Head Neck Surg. 1986;112(1):96‐98. 3940519 10.1001/archotol.1986.03780010098019

[22] Sarna K, Kamau M, Sonigra KJ, Amuti T. Anatomical variations in the origin of the lingual artery in the Kenyan population. Craniomaxillofac Trauma Reconstr. 2021:194338752098310. 10.1177/1943387520983109 PMC8899355 35265271

[23] Balaji S. Reduction glossectomy for large tongues. Ann Maxillofac Surg. 2013;3(2):167. 24205477 10.4103/2231-0746.119230 PMC3814666

